# Does parents’ cognitive ability affect household educational investment? Evidence from Chinese families with left behind children

**DOI:** 10.1371/journal.pone.0286987

**Published:** 2023-06-29

**Authors:** Yuanyuan Wang, Changrong Peng, Shuxun Cai

**Affiliations:** 1 School of Accounting, Hebei University of Economics and Business, Shijiazhuang, China; 2 College of Art, Hebei University of Economics and Business, Shijiazhuang, China; 3 Business School, Hebei University of Economics and Business, Shijiazhuang, China; East China Normal University, CHINA

## Abstract

The large group of left-behind children with the absence of parental accompanying are likely to have serious physical and psychological problems, which may lead to serious public safety and social economic troubles in adulthood. Such unique phenomenon calls us attention on the impact of parents on household educational investment. Based on the data of China Family Panel Studies in 2014, This paper examines the effects of parents’ cognitive ability on household educational investment for their children. The research propositions were tested using multiple regression analysis methods. Results indicate that parents’ cognitive ability can significantly improve the level of monetary and non-monetary investment in education. We also find that compared with their counterparts, the cognitive ability of left-behind children’s parents fails to affect their household educational investment, due to the “parent-child separation effect”. Further analysis shows that improving the regional informatization level of parents of left-behind children can alleviate the “parent-child separation effect”, and finally facilitate cognitive ability’s role in increasing household educational investment. These findings enlighten education policy makers and households a feasible way to alleviate the imbalance and insufficiency of household educational investment among left-behind children families.

## 1. Introduction

The large group of left-behind children families have become a unique phenomenon in China. The so-called Left‐behind children refer to those rural children who are under 18 years of age and are left at home when both or one of their parents migrate to urban area for work [1]. These floating parents (parents of left-behind children) afford their kids through regularly remitting money but without accompanying during daily life. The left-behind children with the absence of parental accompanying are likely to have serious physical and psychological problems [2, 3], which may lead to more serious public safety and social economic troubles in adulthood. There are some left behind children cases in Latin America, Caribbean, Southeast Asia, etc. [4, 5], due to their parents’ transnational migrant work. However, China owns greater left-behind children group than other developing countries. China’s unbalanced regional development and household registration system have made the left-behind children account for about 25.3% of all the children in the whole country by 2015 [6, 7]. Such unique phenomenon calls us attention on the impact of parents on household educational investment. As is known, household educational investment consists of not only monetary investment [4, 5, 8] but also non-monetary investment [9–11]. Monetary educational investment includes extracurricular tutorship, education savings, etc.; and non-monetary educational investment includes daily accompanying regarding homework, participation in school activities, build-up of good parent-child relationships, etc. Therefore, Household educational investment decision is a high information-intensive task, it involves complex information collection and processing which are related to cognitive ability. It has been widely documented in economic literature that human cognitive ability plays a vital role in economic decisions [12–18]. While whether parents’ cognitive ability might affect their household educational investment decisions has yet been explored. Thus, this paper aims to constitute a first step towards addressing this research topic and its underlying mechanism.

Household educational investment on their children can affect the intergenerational human capital accumulation of individual family, and furthermore affect the economic sustainable development of the whole society [19]. The current researches on the influencing factors of family educational investment mainly focus on two aspects, namely, economy and non-economy. With regard to economic factors, some literatures have analyzed the linear relationship and elastic characteristics of educational investment with family income [20, 21]. Other researches on the impact of family financial constraints on children’s education show that the relaxations of family financial constraints in various ways (such as the wealth effect caused by the rising house price) are conductive to increasing the family educational investment and improving children’s educational achievements. With regard to non-economic factors, the previous literatures have investigated the impact of family background, such as Sibship Size [22–24], parents’ social class, parents’ occupation and education level [25, 26], family religious belief, urban and rural background [27, 28], parents’ health status [29], family immigration [30] and other factors on family educational investment. To the best of our knowledge, this is the first paper to examine how parents’ cognitive ability affect their household educational investment on their children.

Since Household educational investment decision involves complex information collection and processing which are related to cognitive ability. We conjecture that cognitive ability, as the final analysis and processing of information, could positively affect household educational investment. However, the impact of parents’ cognitive ability on household educational investment may have heterogeneity between different groups, namely left-behind children families and non-left-behind children families. The parent-child separation of left-behind children family (we regard as “parent-child separation effect” afterwards) makes floating parents (parents of left-behind children) unable to timely collect and process the educational information of their left-behind children. For example, they cannot accurately catch the information of children’s learning performance and psychological status; they are unable to timely and effectively communicate with teachers; and they normally fail to monitor the quality of extracurricular tutorship. Therefore, the unavailability of such important educational information makes the floating parents have much difficulty in passing on their cognitive advantages to the next generation through educational investment, even when they possess high cognitive ability.

China’s left-behind children samples provide us an ideal setting to test the above conjectures. We rely on the data of 2014 China Family Panel Studies (hence, short for CFPS) and document that parents’ high cognitive ability significantly improves household educational investments including both monetary and non-monetary investments. We also show that compared with their counterparts, floating parents’ cognitive ability fails to significantly affect their household educational investments. Further analysis supports the above conjecture of “parent-child separation effect”, by showing that enhanced informatization level of floating parents can alleviate the “parent-child separation effect”, and accordingly facilitate cognitive ability’s role in increasing household educational investment.

The research findings in this paper not only enrich the literature of cognitive economics but also expand the field of educational investment. The results also enlighten education policy makers and households a feasible way to alleviate the imbalance and insufficiency of household educational investment among left-behind children families.

## 2. Literature and hypothesis

Cognitive ability refers to the ability of human brain for information processing, storage and extraction, including memory, reasoning, concentration, language, etc., which represents a person’s ability of knowledge acceptance and information processing. The numerous studies on cognitive economics indicate the significant impact of cognitive ability on individual economic and life decisions, including educational level, health behavior, job performance and income, entrepreneurial behavior, financial investment decision and so on [12–18]. For example, Djankov et al. [13], Levine and Rubinstein [18] found that the stronger the cognitive ability of a person, the more likely he will engage in entrepreneurship. Using European data, Christelis et al. [15] found that the people with higher cognitive ability are more inclined to participate in equity and mutual fund investment. They document that financial market investment is an information-intensive activity, cognition ability reflects individual’s ability of information processing. By taking IQ as the proxy variable of cognitive ability, Grinblatt et al. [17] found that the higher the IQ of the residents, the higher the probability of holding mutual funds and stocks.

Compared with family entrepreneurship and financial decision, the investment decision on children’s education is also an important family economic decision by affecting the intergenerational mobility of family income and social status. The household educational investment decisions in this paper consists of not only monetary investment but also non-monetary investment. Monetary educational investment includes extracurricular tutorship, education savings, etc.; and non-monetary educational investment includes daily accompanying regarding homework, participation in school activities, build-up of good parent-child relationships, etc. Yang and Xu [31] argue that higher information acquisition ability can help family increase educational investment, their data shown that the families with information superiority have higher educational investment level. Therefore, it is expected in this paper that just like family entrepreneurship and financial decisions, family educational investment will also be significantly and positively affected by cognitive ability, thus the first hypothesis is as follows:

**Hypothesis I**: Parents’ cognitive ability could positively affect household educational investment.

However, the effect of parents’ cognitive ability on household educational investment may have heterogeneity between families with left-behind children and their counterparts. The existing literatures show that children’s educational investment under the context of “parent-child separation” has brought the impacts in different directions at the same time. Positive impact mainly refers to the income effect brought by parent-child separation. Some literatures find that the remittance income of immigration workers in Latin America, the Caribbean can help the families of left-behind children out of budget constraints, so as to invest more resources in children’s education [4, 5]. The studies based on the samples of developing countries in Asia also find that the increase in migrant remittance can effectively improve the academic completion rate of left-behind children [32, 33]. However, attentions should also be paid to the negative effects of “parent-child separation” on left-behind children, including the parents’ inability to guide and supervise their children’s studies, and the psychological and physical health problems of left-behind children caused by parent-child separation. For example, the studies based on China’s data find that the time length of left-behind is significantly and negatively correlated to academic achievement and cognitive ability [34].

It is expected that after control the income effect, the parent-child separation effect will make the floating parents unable to timely collect and process the educational information of their left-behind children. For example, they cannot accurately catch the information of children’s learning performance and psychological status; they are unable to timely and effectively communicate with teachers; they normally fail to monitor the quality of extracurricular tutorship. The blocking of these key educational information makes the floating parents even with high cognitive ability unable to pass on their cognitive advantages of collecting and processing educational information to the next generation through educational investment, which is summarized as the following hypothesis:

**Hypothesis II**: Cognitive ability of floating parents does not significantly affect their household educational investment on their left-behind children.

The next realistic question is whether there are feasible ways to increase the family educational investment on left-behind children? Some studies find that cognitive ability can affect the entrepreneurship, financial investment and other economic decision behaviors of individuals through information channels. In fact, providing convenient and efficient ways of information acquisition and searching is beneficial to reduce transaction costs and raise working efficiency and optimize individual economic decisions [35]. Yang and Xu [31] found that the costs of educational information searching and transaction have been greatly reduced for the mothers who using the internet, which finally leads to improvement of family educational investment. While on the contrary, the household educational investment of those families without using the Internet stays at a lower level, due to their inability of reducing information searching cost. In addition, Su et al. [1] found evidence that he children who reported a higher level of parent–child communication reported a higher level of life and school satisfaction and happiness.

If “parent-child separation” may cause the inability of effectively transmitting left-behind children’s educational information to their parents, which is an important reason that floating parents’ cognitive ability fails to play a role, it can be expected that methods of improving information transmission such as internet access will reduce the negative effect of “parent-child separation” and help cognitive ability to play significant role in educational investment. Therefore, the third hypothesis is as follows:

**Hypothesis III**: Improving the information level of floating parents can help their cognitive ability to play a role and as to increase their household educational investment.

## 3. Materials and methods

### 3.1. Data

This paper uses the data of China Family Panel Studies 2014. This survey was launched in 2010 by the China Social Science Research Center of Peking University, is to reflect the changings of China’s society, economy, population, education and other fields by tracing and collecting the micro data of individual, family and community level in the whole country. This article refers to Li et al. [36] and adopts the higher standardized test scores of parental verbal and mathematical abilities as a measure of parental cognitive ability. As only the 2014 CFPS conducted a mathematical cognitive ability test for parents, this paper is mainly based on the third round of CFPS survey conducted in the summer of 2014. In 2014, there were 621 village units, 14,219 families and more than 40,000 family members participating in the survey. In the questionnaire, juvenile is defined as 1–17 years old, so the children in this paper are defined as this age group, usually in primary and secondary school or kindergarten, while those aged 18 and above are defined as adults. The multi-stage and stratified PPS sampling method of the CFPS questionnaire can ensure the representativeness of samples and the credibility of regression results.

### 3.2. Variables

The household educational investment is the dependent variable in this paper. Narrowly, education investment refers to monetary expenditures on education. However, non-monetary resources invested in education also have a significant influence on children’s development [37–40]. Therefore, broadly speaking, family education investment includes not only monetary resources, but also non-monetary resources such as time and spiritual input [41]. The three measures of monetary investment are respectively as: 1) Whether to purchase extracurricular tutorship service (dummy variable); 2) whether to have special education savings for children (dummy variable); 3) amount of extra-curricular tutorship services purchased last year. The two measures of non-monetary investment are: 1) the frequency of checking children’s homework; 2) the frequency of telling stories to children, which can be defined as numerical variables, with their given value ranges of [1, 5] in the questionnaire, indicating the successive increase in the non-monetary (energy and time) investment.

Parents’ cognitive ability as the primary explanatory variable in this paper specially refers to the knowledge and skills acquired through education and experience. For the measurement on cognitive ability, the standardized scores of parents’ cognitive ability is calculated by referring to the methods of Li et al. [36]. The specific calculation method for parents’ cognitive ability is as follows: The first is to calculate the average value and standard deviation of cognitive ability of adults in the same age group by using CFPS data; next step is that the average test scores of the belonging age group are subtracted from the adults’ test scores in verbal and mathematical cognitive abilities, and then divided by the standard deviation of test scores of the belonging age group; finally, for the value of “parents’ cognitive ability”, is to take the higher value of the average standardized scores of parents’ verbal and mathematical cognitive abilities. Obviously, the higher the score, the stronger the cognitive ability of parents.

According to the previous literatures [11, 31] and CFPS questionnaire information, the controlling explanatory variables of education investment include: family net income, the highest education level of parents, the average age of parents, the number of Han nationality, Party members and family size, as well as the province for staying in 2014 and the residential location as urban or rural area. See the following descriptive statistics for the specific meaning of variables. In empirical analysis, the adjustment is based on the CFPS 2014 investigation weight.

### 3.3. Model

We use the following regression setup to test our Hypotheses:

$$Prob(Eduinvest_{i}=1|X)=\Phi (\alpha •cog_{i}+Z\beta +\varepsilon_{i})$$ (1)

$$Eduinvest_{i}=\alpha •cog_{i}+Z\beta +\varepsilon_{i}$$ (2)

Considering the different types of measurement on Educational investment, the econometric model can be set as Probit model (Eq) and OLS model (Eq) respectively, in which *cog*_*i*_ is the cognitive index, *Z* is the control variable, and *X* includes *cog*_*i*_ and *Z*. Here we focus on the direction, magnitude and significance of cog coefficient *α*.

For testing the underlying mechanism, we employ Eq which includes dummy variable of informatization degree into the interaction item by multiplied with cognitive ability index. The significantly positive coefficient *α*_2_ of cross item found in regression results indicates that cognitive ability significantly affects household educational investment through the informatization mechanism.


$$Eduinvest_{i}=\alpha_{1}•cog_{i}+\alpha_{2}•infor\_dummy_{i}•cog_{i}+Z\beta +\varepsilon_{i}$$ (3)

## 4. Results

### 4.1. Descriptive statistics

The definition and descriptive statistical characteristics of variables are listed in Table 1. The last column shows the difference between families of left-behind children and non-left-behind children. Obviously, parents’ cognitive ability of left-behind children is significantly lower than non-floating parents’; household educational investment of left-behind children families are significantly lower than that of non-left-behind children, for example, the respective investments in extra-curricular class are 191.486 yuan and 388.679 yuan, which show a big gap between the two groups in family education investment; finally, the income, age and education level of left-behind children families are obviously at a disadvantage.

**Table 1:** Variable descriptive statistics.

Variable	N	Mean	Mini	Max	Differences
Whether to purchase extra-curricular tutorship	6832	0.129	0	1	-3.113***
Whether to have educational savings	6832	0.254	0	1	-1.701*
Amount of extra-curricular tutorship expenses (thousand yuan)	6832	0.360	0.000	60	-3.056***
Frequency of checking children’s homework	3818	3.389	1	5	-8.218***
Frequency of telling stories to children	2121	2.878	1	5	-2.167**
Cognitive ability	6596	0.406	-2.816	2.768	-8.876***
Verbal cognitive ability	6596	0.445	-2.999	2.488	-8.323***
Mathematical cognitive ability	6596	0.456	-2.634	3.048	-8.458***
Cognitive ability (in 2010)	5979	0.351	-2.456	2.192	-6.582***
Verbal cognitive ability (in 2010)	5979	0.404	-2.652	2.194	-6.281***
Mathematical cognitive ability (in 2010)	5979	0.376	-2.283	3.038	-6.278***
Family net income (million yuan)	6331	0.054	0.000	4.073	-4.232***
Parents’ average age	6832	40.46	17	87	5.009***
Highest educational level among parents	6464	2.498	1	8	-10.555***
Han nationality of all parents	6832	0.730	0	1	-1.539
One of the parents is a party member	6486	0.016	0	1	-0.966
Number of family members	6832	5.332	2	17	2.205**
Whether to live in city	6796	0.466	0	1	-13.333***
Province code	6832	36.515	11	65	2.545**

Differences (in the last column), the differences between families of left behind children and their counterparts.

^a^ *, ** and *** indicate statistical significance at the 10%, 5% and 1% levels.

**Fig 1:**
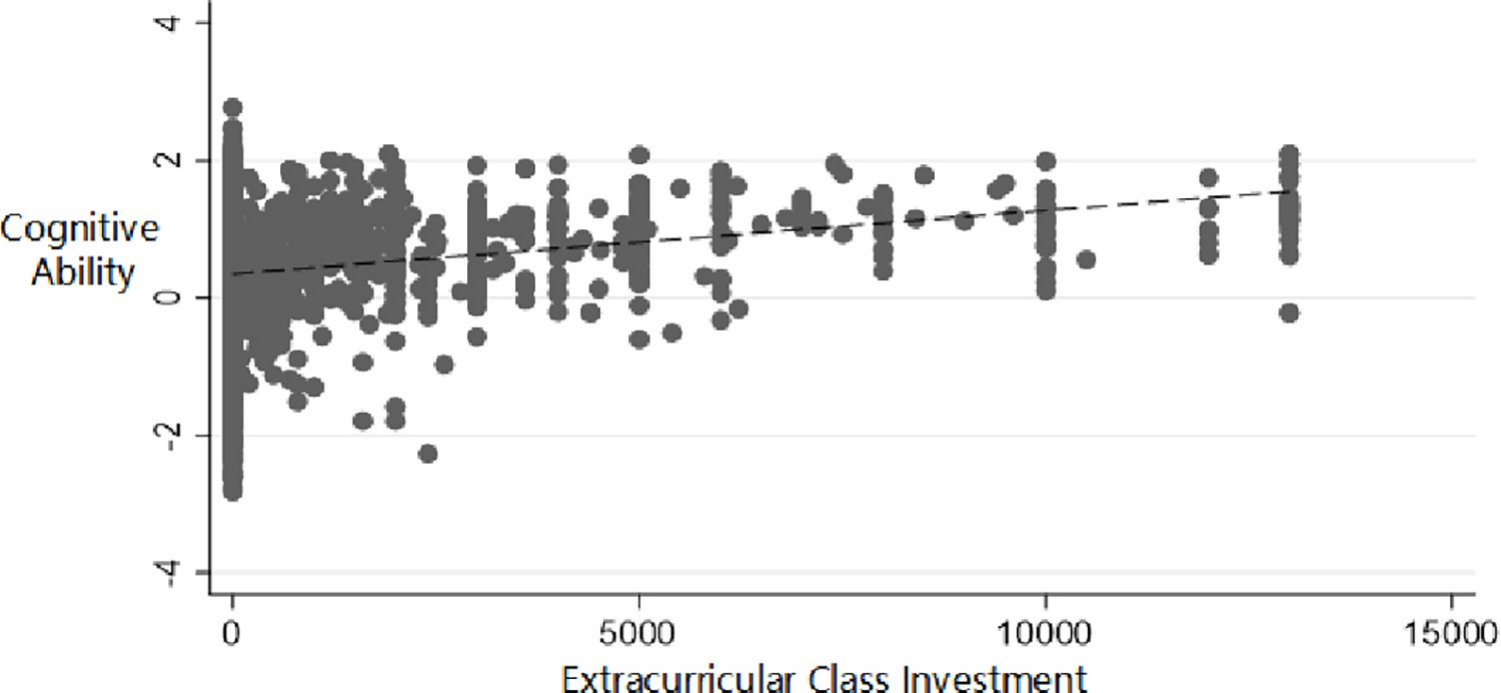
Positive correlation between cognitive ability and extracurricular class investment. (A) The amount of extracurricular auxiliary fees here has been abbreviated by 5%. (B) The amount of family tutoring fees increases with the improvement of parents’ comprehensive cognitive ability.

Without the control on other variables, the relationship between the amount of extra-curricular tutorship expenses and the cognitive ability is shown in Fig 1. It can be intuitively found that the amount of extra-curricular tutorship expenses increases with the improvement of parents’ cognitive ability.

### 4.2. Regression results

#### 4.2.1. Benchmark results

Probit regression is conducted according to model (1). From Table 2, it can be seen that after controlling the variables such as family net income, educational level, average age of parents, all parents as Han nationality, one of the parents as Party member, the number of family members, as well as the province for staying in 2014 and residential location as urban or rural area, the regression results of each column show that parents’ cognitive ability can significantly increase the possibility of household educational investment on their children. For example, every 0.1 increase in cognitive ability will make the probabilities of purchasing extra-curricular tutorship and conducting educational savings increase by 1.11% and 1.43% respectively. Therefore, the regression results in Table 2 prove that higher cognitive ability can significantly increase monetary investment probability on their children’s education (measured by the purchase of extracurricular counseling services and the existence of special education savings), thus verifying Hypothesis I.

**Table 2:** Overall impact of cognitive ability on household educational investment.

Variable	Probit		OLS		
	Whether to purchase tutorship	Whether to have educational savings	Extracurricular tutorship expenses	Frequency of checking homework	Frequency of telling stories
	(1)	(2)	(3)	(4)	(5)
Cognitive ability	0.111**	0.143***	0.102***	0.264***	0.142**
	(0.047)	(0.037)	(0.031)	(0.045)	(0.068)
Controls	Yes	Yes	Yes	Yes	Yes
Sample size	5,624	5,624	5,626	3,265	1,837
R^2^	-	-	0.147	0.078	0.121

^a^ The superscripts “* “, “**” and “***” respectively indicate the statistical significance at 10%, 5% and 1% levels, with the robust standard error in brackets.

^b^ For brevity, control variables are omitted from this table and the followings.

OLS regression is conducted according to model (2). The OLS regression results are consistent with Probit regressions. Column (3) shows the impact of parents’ cognitive ability on the amount of extra-curricular tutorship expenses, column (4) to column (5) shows the impact of parents’ cognitive ability on “the frequency of talking to children about school” and “the frequency of checking children’s homework”. It can be seen that all of these columns show significantly positive effect of parents’ cognitive ability on monetary and non-monetary educational investment. Thus, these results further verify our Hypothesis I.

#### 4.2.2. Heterogeneity analysis

We run the following regressions to examine the heterogeneity in the influence of parents’ cognitive ability on their children’s educational investment between left behind children families and their counterparts. In this paper, the negative answer to the question that “whether the parents live with children?” will be defined as the families of left-behind children, since there is no direct definition on left-behind children in CFPS data. As shown in Table 3, Panel A lists regression results of the families of non-left-behind children and Panel B shows the regression results of the families of left-behind children. From the Probit regressions, it can be seen that, compared with the families of left-behind children, the cognitive ability of parents who living with their children can improve the possibility of children’s educational investment. While, the cognitive ability of floating parents has no significantly positive impact on the probability of their children’s educational investment, thus primarily verifying the hypothesis II.

**Table 3:** Heterogeneous impact of cognitive ability on household educational investment.

**Panel A: Non-left-behind Children samples**	**Probit**		**OLS**		
	**Whether to purchase tutorship**	**Whether to have educational savings**	**Extracurricular tutorship expenses**	**Frequency of checking homework**	**Frequency of telling stories**
	(1)	(2)	(3)	(4)	(5)
Cognitive ability	0.136***	0.164***	0.119***	0.263***	0.165**
	(0.051)	(0.041)	(0.038)	(0.052)	(0.071)
Controls	Y	Y	Y	Y	Y
Sample size	4,844	4,844	4,846	2,583	1,667
R^2^	-	-	0.146	0.076	0.129
**Panel B: Left-behind Children samples**	**Probit**		**OLS**		
	**Whether to purchase tutorship**	**Whether to have educational savings**	**Extracurricular tutorship expenses**	**Frequency of checking homework**	**Frequency of telling stories**
	(1)	(2)	(3)	(4)	(5)
Cognitive ability	0.007	-0.004	0.010	0.322***	0.088
	(0.123)	(0.091)	(0.048)	(0.087)	(0.212)
Controls	Y	Y	Y	Y	Y
Sample size	655	759	779	682	170
R^2^	-	-	0.257	0.179	0.316

^a^ The superscripts “* “, “**” and “***” respectively indicate the statistical significance at 10%, 5% and 1% levels, with the robust standard error in brackets.

The heterogeneous impact of parents’ cognitive ability on children’s educational investment is further investigated by OLS regressions. In the OLS regressions, educational investment includes monetary investment (amount of extracurricular tutorship expenses) and non-monetary investment (such as the frequencies of checking children’s homework and telling stories to children). The OLS regression results show that compared with their counterparts, the floating parents’ cognitive ability doesn’t have significant impact on household educational investment nearly in all measures (One exception is “checking homework” in column 4 Panel B, which is probably because in China checking homework is an obligatory task required by school, thus parents may entrust other family members to help parents with homework checking). Thus, the above results support our Hypothesis II.

#### 4.2.3. Mechanism analysis

In this section, we test the underlining mechanism for the effect of cognitive ability on household educational investment. If “parent-child separation” may cause the inability of effectively transmitting left-behind children’s educational information to their parents, which is an important reason that their cognitive ability fails to play a role, it can be expected that methods of improving information transmission such as internet access will reduce the negative effect of “parent-child separation” and help cognitive ability to play significant role in increasing educational investment. To test this mechanism, the indicator of informatization degree at district-county level is firstly constructed by parents accessing to Internet or not. The districts and counties higher than the mean value of national average informatization degree are defined as the high-informatization district-county dummy, which is then multiplied by cognitive ability to obtain the cross item of “Cognitive ability ╳ informatization degree”.

As shown in Table 4, among the regression results of information mechanism for the impact of parents’ cognitive ability on children’s educational investment, the full-sample results are shown in column (1)(2)(3), while the sub-sample results of the families of left-behind children are shown in the column (4)(5)(6). It can be seen that compared with the parents in low-informatization regions, the parents in high-informatization regions will increase their children’s educational investment as cognitive ability increasing. In particular, the impact of higher informatization on families of left-behind children is even greater and more significant, for example, for the samples of left-behind groups, the joint effect of cognitive ability and high informatization on increasing of the amount of extracurricular tutorship is 35.4%, comparing with 25.2% in full sample (see the column 3 in Table 3); similarly, for the samples of left-behind groups, the joint effect of cognitive ability and high informatization on parents’ non-monetary investments is also higher than that of the full sample (see column 4 and 5). These results support our hypothesis III.

**Table 4:** Influential mechanism of cognitive ability on household educational investment.

	OLS: Full samples			OLS: Left-behind Children samples		
	Extracurricular tutorship expenses	Frequency of checking homework	Frequency of telling stories	Extracurricular tutorship expenses	Frequency of checking homework	Frequency of telling stories
	(1)	(2)	(3)	(4)	(5)	(6)
Cognitive ability	0.001	0.196***	-0.005	-0.094*	0.192**	-0.191
	(0.031)	(0.053)	(0.081)	(0.056)	(0.092)	(0.231)
Cognitive ability╳informatization degree	0.252***	0.174**	0.356***	0.354***	0.482***	0.868**
	(0.065)	(0.069)	(0.108)	(0.135)	(0.142)	(0.389)
Controls	Y	Y	Y	Y	Y	Y
Sample size	5,626	3,265	1,837	779	682	170
R^2^	0.150	0.081	0.129	0.270	0.198	0.352

^a^ Probit regression results do not converge, so the results are omitted here.

^b^ The superscripts “* “, “**” and “***” respectively indicate the statistical significance at 10%, 5% and 1% levels, with the robust standard error in brackets.

### 4.3. Robustness tests

It is important to point out that the impact of parents’ cognitive ability on children’s educational investment may have the endogenous problems. Considering that the explanatory variables have been controlled as much as possible in regression analysis, this endogeneity mainly comes from the reverse causality, that is, not only the family educational investment affected by cognitive ability, but also parents’ family educational investment as an important economic behavior of the family are likely to reshape and enhance their cognitive ability in its process and results. In order to eliminate the endogenous problems caused by the reverse causality, we adopt a similar method used in Heineck and Anger [18] and Li Tao et al. [36]. The cognitive ability of CFPS data in 2010 is used in this paper to replace the corresponding variable in 2014, that is, overcome the reverse causality bias by investigating the impact of parents’ cognitive ability in 2010 on family educational investment in 2014. As shown in Table 5, after alleviating the reverse causality, cognitive ability can still significantly improve the household educational investment, and the regression results have no significant change comparing to the Table 2.

**Table 5:** Endogenous test results.

Variable	Amount of extracurricular tutorship	Frequency of checking homework	Frequency of telling stories to children
	(1)	(3)	(4)
Cognitive ability in 2020	0.160***	0.187***	0.155
	(0.053)	(0.064)	(0.104)
Controls	Y	Y	Y
Sample size	3,063	2,014	941
R^2^	0.217	0.079	0.151

^a^ The superscripts “* “, “**” and “***” respectively indicate the statistical significance at 10%, 5% and 1% levels, with the robust standard error in brackets.

In addition, in order to conduct robustness tests, we use alternative measures for our main variables. For example, “whether parents and children live together” is redefined as “whether parents take care of children at night” or “whether they have lived with their parents for more than 11 months in the past 12 months”. “whether surf the Internet” is redefined as “the evaluation on subjective importance of internet information” to reconstruct the district-county informatization degree. The above results hold when we use alternative variables to run new regressions.

## 5. Conclusions

The main purpose of this study was to investigate whether and how parents’ cognitive ability affects household educational investment on their children. This study is related to two strands of literature. One strand is the literature on determinants of household educational investment, such as Sibship Size, parents’ social class, parents’ occupation and education level, family religious belief, urban and rural background, parents’ health status, family immigration and etc. [22–30]. The other strand is the literature on effects of cognitive ability on economic decisions including individual educational achievement, health behavior, job performance and income, entrepreneurial behavior, financial investment decision etc. [12–18]. To the best of our knowledge this paper is the first study to combine these two strands of literature, and investigate the heterogeneity effect of parents’ cognitive ability on household educational investment (includes both of monetary and non-monetary investment) and exploit the underlying mechanism.

The findings of the present study confirmed a positive effect of parents’ cognitive ability on household educational investment. This result enlightens education policy makers, since the current educational system in China more focus on the improvement of educational investment from the teacher-student side, but ignores the important role of parents in education. The lack of guidance and training on parents in household educational investment decision making has resulted in many problems in some families. Thus, the top-down establishment and improvement of parent training system can help to enhance the efficiency of household educational investment.

The further analysis of this study showed floating parents’ cognitive ability does not significantly affect their household educational investment due to the “parent-child separation effect”. Internet access for floating parents can help cognitive ability to play a role to increase educational investment. China’s rapid development and household registration system have made the left-behind children account for about 25.3% of all children in the whole country. However, in practical operation, it is very difficult to solve the problem of the absence of parents’ educational investment by forcing parents not to go out for working or requiring them to take their children to their working city. Thus, solving such problem becomes very difficult. Considering that the policy effects of traditional solutions such as the ablation of household registration system or the increase in government education supply are weakening, the conclusions of this research provide a feasible way to alleviate the absence of educational investment at low cost, that is, by popularizing and improving the informatization of parents of left-behind children. Enhancement of the information transmission between parents and children can reduce the parents’ cost of information acquisition, so as to help them to make better educational investment decisions. The improvement of informatization is beneficial to make up for the short board of parents’ cognitive ability and enhance the education investment of parents, especially the parents of left behind children.

In addition, under the global outbreak of Corona Virus Disease 2019 (COVID-19) from the end of 2019, network teaching as a new teaching mode of schools implemented in many countries and regions, including China. The network teaching lacks the teacher-student face to face interactions such as communication, supervision and instant feedback, which make it different from the traditional face-to-face teaching mode. To ensure the quality of network teaching in primary and secondary schools, parents not only invest on hardware facilities such as computer, camera, microphone and high-speed network, but also have to invest more time, energy and intelligence to supervise and guide their children’s studies. This new teaching mode makes higher requirement on parents’ cognitive ability. Therefore, in the special period of home quarantine and network teaching, this public emergency has made the role of parents’ cognitive ability as a more important influencing factor on family educational investment and children school achievement, future studies are needed for this new phenomenon.
